# Integrated obesity care management system -implementation and research protocol

**DOI:** 10.1186/1472-6963-7-163

**Published:** 2007-10-10

**Authors:** Jean-Patrice Baillargeon, André Carpentier, Denise Donovan, Martin Fortin, Andrew Grant, Judith Simoneau-Roy, Denise St-Cyr-Tribble, Mariane Xhignesse, Marie-France Langlois

**Affiliations:** 1 Division of Endocrinology, Department of Medicine, Université de Sherbrooke, Sherbrooke, Québec, Canada; 2 Department of Family Medicine, Université de Sherbrooke, Sherbrooke, Québec, Canada; 3 Unité de médecine de famille, Centre de Santé et de services sociaux de Chicoutimi, Chicoutimi, Québec, Canada; 4 Department of Biochemistry, Université de Sherbrooke, Sherbrooke, Québec, Canada; 5 Department of Pediatrics, Université de Sherbrooke, Sherbrooke, Québec, Canada; 6 École des sciences infirmières, Université de Sherbrooke, Sherbrooke, Québec, Canada

## Abstract

**Background:**

Nearly 50% of Canadians are overweight and their number is increasing rapidly. The majority of obese subjects are treated by primary care physicians (PCPs) who often feel uncomfortable with the management of obesity. The current research proposal is aimed at the development and implementation of an innovative, integrated, interdisciplinary obesity care management system involving both primary and secondary care professionals.

**Methods:**

We will use both action and evaluative research in order to achieve the following specific objectives. The first one is to develop and implement a preceptorship-based continuing medical education (CME) program complemented by a web site for physicians and nurses working in Family Medicine Groups (FMGs). This CME will be based on needs assessment and will be validated by one FMG using questionnaires and semi structured interviews. Also, references and teaching tools will be available for participants on the web site. Our second objective is to establish a collaborative intra and inter-regional interdisciplinary network to enable on-going expertise update and networking for FMG teams. This tool consists of a discussion forum and monthly virtual meetings of all participants. Our third objective is to evaluate the implementation of our program for its ability to train 8 FMGs per year, the access and utilization of electronic tools and the participants' satisfaction. This will be measured with questionnaires, web logging tools and group interviews. Our fourth objective is to determine the impact for the participants regarding knowledge and expertise, attitudes and perceptions, self-efficacy for the management of obesity, and changes in FMG organization for obesity management. Questionnaires and interviews will be used for this purpose. Our fifth objective is to deliver transferable knowledge for health professionals and decision-makers. Strategies and pitfalls of setting up this program will also be identified.

**Conclusion:**

This project is relevant to health system's decision-makers who are confronted with an important increase in the prevalence of obesity. It is therefore critical to develop strategies allowing the management of obesity in the 1^st ^line setting. Results of this research project could therefore influence health care organization in the field of obesity but also eventually for other chronic diseases.

## Background

### Obesity – a major health issue

Obesity is a recognized major public health problem identified as an epidemic by the World Health Organization [1]. As of 2001, nearly 50% of the Canadian population was reported as being overweight or obese [2]. The prevalence of childhood obesity in Canada has tripled between 1981 and 1996 to reach about 12% [2,3] and there is strong evidence that parental obesity increases the risk of offspring obesity [4]. The direct cost of obesity in Canada was recently estimated at 4.3 billion dollars, in addition to 5.3 billion dollars related to sedentary lifestyle: this represents 4.8% of our health care system's budget [5].

It is well known that obesity is associated with increased risk for cancer, type 2 diabetes, hypertension, hyperlipidemia and atherosclerosis, resulting in an increased mortality rate [6,7]. Moreover, there is an increased mortality rate in adults who were obese as children, regardless of their adult weight [8]. Therefore, identification and management of obesity must start during childhood and adolescence. Nonetheless, moderate weight loss in obese subjects has been proven to markedly decrease the incidence of diabetes, lower blood pressure, improve lipid profile and decrease mortality [9-13].

### Management of obesity – recommendations

The recently published guidelines on the management of obesity [14-16] include the following essential components: 1) setting realistic weight loss goals i.e. 5 to 10% weight loss at a rate of 1 pound per week or less; 2) continuous support with frequent visits; 3) emphasis on the long-term maintenance of weight loss; 4) individualized, moderate caloric restriction; 5) individualized regular physical activity; 6) self-monitoring and maintenance of records; 7) individualized behavioural strategies such as stimulus control and stress management; 8) support groups; 9) participant informed consent; and 10) the use of pharmacotherapy and/or surgery in selected cases. The NIH and Canadian Obesity Guidelines [17] strongly encourage the establishment of an interdisciplinary team or, when not possible, referral of patients to such a team.

The Canadian obesity guidelines give evidence-based guidelines for the treatment of pediatric and adolescent obesity. Based on expert opinion [18], treatment guidelines have focused on lifestyle intervention for pediatric obesity. Weight loss is encouraged only in severely obese children, and maintenance of body weight with continued linear growth, accompanied by development of healthier lifestyle, should be the ultimate goal [19]. Evidence for the efficacy of family-based behavioral approaches in obese adolescents are only available in well-controlled research studies and long-term outcome data are very limited [20,21]. Nevertheless, such approaches seem to be at least as effective as in adults [22,23]. Unfortunately, specific interventions are initiated by primary care physicians in less than 20% of overweight pediatric patients [24], probably because this approach is labor intensive and requires specific knowledge that is not yet easily translated by expert obesity physicians to primary care givers.

### Organization of primary health care services in Quebec, Canada

The implementation of Family Medicine Groups (FMGs, *Groupe de Médecine de Famille*), is the major organizational change to have occurred in primary health care services in Quebec in response to the 2000 Clair Commission [25]. Improved accessibility and continuity, interdisciplinarity and patient registration are some of the central principles of this reorganization. Within the FMG context, family physicians are called upon to work closely with nurses to deliver more comprehensive care. This provides further opportunities to improve care for specific conditions that call for interdiscliplinary interventions and warrant lifestyle modifications. Obesity represents a special challenge that is increasingly addressed in some FMGs [26].

The province of Quebec's Integrated University Health Networks, known as the RUIS (*réseaux universitaires intégrés de santé*), provide the opportunity to access FMGs within the entire territory of a given RUIS. The *Université de Sherbrooke *RUIS caters to a population of over 1 million people and encompasses a vast territory including the Sherbrooke area, the Montérégie area, and the distant Saguenay/Lac St-Jean region (for teaching and research). The *Université de Sherbrooke *RUIS therefore provides a broad geographical representation of FMGs and an exceptional opportunity to study the proposed implementation of our program in a variety of proximity settings. This is expected to enhance the applicability of results to many other different settings throughout Canada.

Considering the growing prevalence of obesity in Canada, the majority of patients should be managed by primary care givers. Unfortunately, primary care physicians (PCPs) often feel that obesity treatment efficacy is poor [27]. As a result, obesity tends to be neglected when compared to other chronic conditions like hypertension and diabetes. When auditing medical records from PCPs, obesity is definitely underreported and recommendations of weight control intervention are reported even less [28]. This only highlights the need for major changes in medical practice regarding this important health problem.

### Interdisciplinary approach to obesity – previous and preliminary results

Considering the significance of obesity and existing recommendations, our group implemented an innovative interdisciplinary approach to obesity care management in 2001. Managed care is an evolving principle stemming from the 1990's reform. According to Graber O'Brian [29], interdisciplinary and collaborative care are important parts of managed care. Our team includes a nurse-clinician, a nutritionist, a psychologist, a kinesiologist and several endocrinologists. The team is coordinated by the nurse-clinician and offers a variety of behavioural approaches to step-wise lifestyle modification using a series of 24 weekly group seminars and individual consultations with health professionals every 6 weeks, or as needed. There are no out of pocket costs to the patient in our obesity care management system. To our knowledge, this system is unique in Canada and provides a unique opportunity to evaluate the effectiveness of implementing an interdisciplinary approach to the management of obesity without the inherent patient selection biases of specialized interventions requiring fee-for-service.

A retrospective analysis of > 130 obese patients managed by our interdisciplinary team in 2002 and 2003, revealed that 35% had lost 5% or more of their initial body weight after 6 months in our program (unpublished data). Also, preliminary data from a randomized controlled study in 60 patients with metabolic syndrome shows a mean weight loss of 4.8% at 6 months in our intervention group compared to 0.8% in the control group that was followed by PCPs (data on 41/60 patients) [30]. These results are comparable with those of proven effective interventions to prevent type 2 diabetes [9,10].

### Preceptorships and use of electronic tools in obesity care management

Access to our program is unfortunately limited due to financial constraints, with a 2 year waiting list for admission of new patients. In the fall of 2003, we developed, in collaboration with the Continuing Medical Education (CME) Center of the *Université de Sherbrooke*, preceptorships in obesity as a mean of engaging PCPs in obesity management to: 1) improve their expertise/attitudes toward obesity care and 2) implement more effective obesity management in their milieu. These initial preceptorships consisted in a full day of interactions between small groups of PCPs (3 to 6) and the personnel of our clinic.

Preceptorship evaluations by PCPs were extremely positive, with twenty two out of 23 individuals who would strongly recommend the program to a colleague. What was most appreciated by participants was the privileged contact both with the obesity clinic health professionals and with patients throughout their consultations. Theoretical notions were reviewed then exemplified through clinical exposure (seeing real patients). This is reported in the literature[31] to effect change in professional practice, and, on occasion, health care outcomes. Indeed, variables that appear to have a positive effect in terms of enhancement of physician performance are "the degree of active learning opportunities, learning delivered in a longitudinal or sequenced manner and the provision of enabling methods to facilitate implementation in the practice setting" [31].

In refining our preceptorships, we identified that coupling the preceptorship with the establishment of a virtual learning community as well as specific tools for the practice setting are key ingredients identified to effect change in practice. Internet based communication, accessed at one's own time in the clinic or at home, or used for same-time meetings, is transforming the continuing information environment. *e*Learning is becoming increasingly popular and was proven to be at least as effective as classical CME to improve knowledge, and may initiate changes in medical practice [32]. The *Université de Sherbrooke *CASSIS (*Centre d'Applications du Savoir et du Soutien en Informatique Santé *– Centre for Knowledge Applications and Health Informatics Support) research collaboration has developed an innovative life-long learning web based architecture in association with its current on-line educational activities with health professionals (certificate program in Health Informatics; Quebec virtual campus; projects in virtual communities). We saw the opportunity for this web-based learning structure to be implemented in tandem with preceptorships in the proposed project [33].

The architecture supports different dimensions. Different theoretical considerations, from cognitive, educational, organizational, sociological, technical and other disciplines underlie the creation of this architecture. The various actors, specialists and community care givers, physicians, nurses, nutritionists and other team members, patients and their families make up a learning community. The term "virtual learning community" has recently gained common use where supported by web based communications. Virtual learning communities are defined by the cooperative process and are not restricted to structured courses. They follow integrative global objectives and require facilitation and technical expertise if they are to be sustained. Potential advantages of an *e*Learning community include a more fluid communication across the different actors resolving of complex situations; flexibility with respect to different working environments and ethos [34-40].

### Hypothesis

We hypothesize that CME based on preceptorships in combination with electronic tools (web site and virtual community) for networking primary care (FMGs) and secondary care health professionals will (a) enhance FMG team members expertise and knowledge; (b) modify attitudes and perceptions with respect to obesity and its treatment; (c) increase the feeling of self-efficacy of FMG professionals toward the management of obesity; and (d) foster the implementation of nurse-coordinated team management for obesity in primary care settings, and eventually improve access to quality support resources for PCPs and their teams, as well as for overweight or obese individuals.

### Objectives and specific aims

The current research proposal aims to further develop, implement, and evaluate the combined preceptorships/eLearning network for FMGs throughout the RUIS, and to set up an interdisciplinary obesity primary care system. This specific aim is in accordance with many thematic areas identified as being of high priority by the Canadian Institutes of Health Research (CIHR), namely: (1) Linking care across place, time and settings; (2) Linking public health to health services; (3) Nursing leadership, organization and policy; and (4) Timely access to quality care for all. System support for managing obesity and overweight is also one of the three sub-themes identified by CIHR.

**Figure 1:**
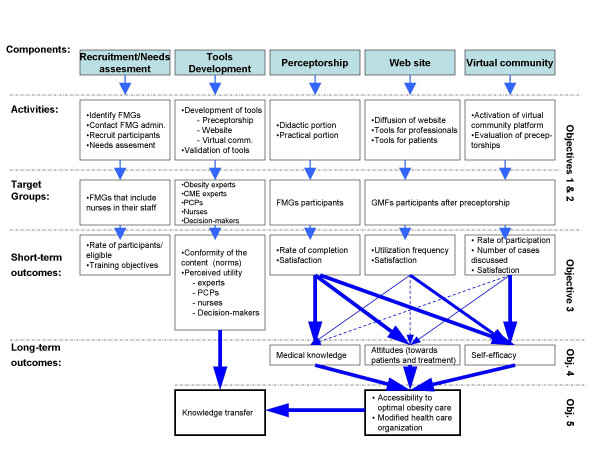
Enhancement of integrated, interdisciplinary, primary care management of obesity.

Figure 1 represents all aspects of our program with related activities, target groups as well as short-term and long-term outcomes. Each step or outcome level of our research program is associated with the following specific objectives:

1. To further develop and implement a preceptorship-based CME for FMG teams, including online education and teaching materials to support nurse-based management programs in primary care settings;

2. To set up aa a collaborative intra/inter-regional interdisciplinary network, including a virtual-community platform, to enable on-going expertise update and networking for FMG teams;

3. To evaluate implementation of this integrated obesity care system by assessing the:

a. ability of the preceptorship structure to train 8 FMG teams during the research program,

b. accessibility and utilization of the website and virtual community following preceptorships for FMG team members, and

c. satisfaction of PCPs, nurses, other FMG team members and obesity specialists, with respect to preceptorship training and network system;

4. To assess the impact of the preceptorships and network system on obesity primary care delivery, from the perspective of participating FMG team members and with respect to:

a. knowledge and expertise both for adult and pediatric populations,

b. attitudes toward patients and treatment effectiveness, and

c. perception of self-efficacy;

5. To deliver transferable knowledge on:

a. the potential pitfalls and strategies of setting up preceptorships/website/virtual community network systems to enhance integrated interdisciplinary obesity primary care management,

b. the progressive development of appropriate performance indicator sets assessing obesity care accessibility and delivery, using a Canadian recommended framework.

## Methods

### Research design

Quebec health services reform considers support of primary care professionals as an integral part of the responsibility of second line professionals [25]. Accordingly, this research offered the means of supporting primary health care as part of the routine professional activity of second line caregivers. The proposed study relies on a mixed research design, of action research as well as focused evaluation. Action research and focus evaluation are both part of the collaborative research paradigm. According to Balfour and Clark [41] inquiry involving practitioner's knowledge of patients, professional and environmental factors in the context of care delivery may require action research-based evidence. In the same train of thought, Patton [42] mentions that program assessment may largely profit from the use of focused evaluation utilization. Thus both research designs facilitate the inclusion of all relevant stakeholders in the evaluation process.

For the above mentioned reasons, we proposed an action-based research design under objectives 1 and 2 [43]. Action-research enabled us to respond to needs as they occured during the development of our intervention. Such responsiveness is critical to the success and the durability of the project. The progressive iterations of design and evaluation are also essential for successful electronic tool development [44].

The evaluative approach under objectives 3 and 4 was utilization-focused [42]. As project managers, we are the primary users of the evaluation results alongside our decision-maker partners. In focusing on the needs of actual primary users, results are expected to be of interest to other second line professionals with respect to primary care support activity planning as well as to decision-makers wishing to adapt a model such as ours to other situations. The logic model, as it stands in Figure 1, divides the project into several components: Recruitment, Tool development, Preceptorship, Web site and Virtual Community program. Components of the model were further elaborated and indicators or standards of activities and outcomes agreed upon with our partners [45].

### Participants

Under objectives 1 and 2: Development and implementation of a preceptorship, web site and virtual community, we first identified participating FMGs within the RUIS (clinical and teaching). A FMG was eligible to participate in the proposed study if at least one PCP and one nurse were interested. We plan to recruit a total of 8 FMGs; appropriate regional authorities have approved the proposed research project. For each identified FMG, PCPs and nurses will be informed of the study and invited to participate. All interested FMG team members will be recruited with a maximum of 6 participants per FMG, 48 participants being the capacity limit of our preceptorship program.

Upon recruitment, participants will complete a needs-assessment questionnaire regarding obesity management and be asked to identify their expectations. An additional questionnaire will review their past experience with respect to obesity management. Preceptorships will be based on the existing format, i.e. a combination of theoretical and practical CME, and will include common and nurse- or PCP-specific sessions.

### Ethical considerations

The proposed research was reviewed and approved by the appropriate institutional Research Ethics Review Boards according to applicable legislations and the Tri-Council Policy Statement. Research participants will be duly informed and consent will be obtained in writing prior to participation. Ethical considerations raised by the proposed research are mainly confidentiality issues. All data will be coded, archived for at least 5 years and then destroyed.

### Research tools

A scientific committee will be mandated to recommend modifications to the existing preceptorship program and web site tools based on needs-assessment evaluation and review of participants' experience, in accordance with norms of clinical care and perceived utility. This committee will include obesity experts, CME experts, PCPs, nurses and our ADRLSSSE (*Agence de développement de réseaux locaux de services de santé et de services sociaux de l'Estrie*) decision-maker co-investigators. Pursuant to our action-research model, decision-makers will be involved throughout program development and implementation, as well as the evaluation process.

We will also develop a website with online tools and virtual community network based, in part, on the input of the above-mentioned scientific committee. Network participants will gather through monthly web-based meetings (specific subject overviews, discussion of difficult cases and journal club). The proposed network will be supported by online educational material, specifically designed and aimed at physicians, nurses and patients. FMG nurses will access all necessary resources to implement group seminars and other nurse-based interventions in their milieu through the network. Internet communications will provide for immediate feedback to and from the coordinating team (discussion forum). We have special expertise in the development of online education and the methodologies of virtual communities working with several partners including the Institute for Knowledge Innovation and Technology.

The association of a virtual learning community and a clinical preceptorship into a unified activity is innovative and should be conducive to changes in practice. All dimensions of the lifelong learning architecture [33] apply to this project.

Tools developed will first be validated with one selected FMG and focus groups will subsequently identify any pitfalls and irritating factors that could undermine the implementation of the program. Once again, decision-makers will be invited to actively participate in focus groups and the evaluation process. When program development is completed and evaluation tools developed, FMG teams will be invited to attend preceptorships based on their availability. All members of the same FMG will participate together free of charge.

As soon as FMGs begin their participation, we will conduct a prospective evaluation of the implementation of the preceptorship and network system. Innovative web-based tools will be used for this purpose, as well as more traditional methods. Since evaluation is crucial to the applicability and transferability of results under objectives 4 and 5, decision-makers will be involved early and actively throughout the process. This should ensure that decision-makers are active members of the research team and as such increase the probability that they will use the study results.

Specific research tools for each objective are summarized in Table 1, which includes corresponding indicators and variables as well as the sources that will be used to obtain required data. We will not detail each of our methods in order to improve the readability of this paper. However, detailed methodologies will be made available to the reader on request by contacting the corresponding author.

**Table 1:** Summary of Methodology and Approach

**Objectives**	**Indicators or variables**	**Sources**
**1. **Develop and implement a preceptorship-based CME for FMG teams	a. Perceived pitfalls to implementation of the program	• Focus groups
**2. **Set up aa a collaborative intra/inter-regional interdisciplinary network		
**3. **Evaluate implementation of this integrated obesity care system:		
**3a) **ability of preceptorship structure to train 8 FMG teams during the research program	a. Proportion of candidates who agree to participate b. Proportion who participates in the preceptorship c. Resources needed for each preceptorship	• Data from the regional health agency • Administrative data from the project team • Budget and administrative data from the project team
**3b) **accessibility and utilization of the website and virtual community following preceptorships for FMG team members	a. Number of times participants access the website b. Length of time spent and the number of times individual tools are accessed c. Perceived utility of the tools in general d. Most helpful tools e. Obstacles and facilitating factors in using the website f. Resources needed to maintain the website g. Team members participation to the virtual community h. Degree to which the virtual community meets physicians' needs i. Resources needed to support the virtual community j. Degree to which participants perceived they achieve optimal obesity care delivery k. Perceived utility of each tools to optimal care delivery	• Web logging tools • Web-based questionnaires • Telephone and person-to-person structured interviews • Clinical vignettes and web-based learning tools • Web-based anonymous partial patient records • Budget and administrative data from the project team • Secondary care professionals' logbooks
**3c) **satisfaction of FMG team members and secondary care professionals	a. Participants expectations b. Participants satisfaction with the preceptorship c. Participants satisfaction with the website d. Participants satisfaction with the virtual community e. Suggested improvements by participants f. Secondary care team members satisfaction with preceptorship, web-site and virtual community g. Secondary care team members ease with this method of sharing expertise and patient care h. Suggested improvements by secondary care team members	• Traditional and web-based questionnaires • Telephone and person-to-person structured interviews • Focus groups with selected participants • Telephone and person-to-person structured interviews of secondary care team members • Focus groups with selected secondary care team members
**4. Assess the impact of the preceptorships and network system on obesity primary care delivery, related to participating FMG team members:**	Number of patients managed in the network	Web-based participants log-book
**4a) knowledge and expertise**	a. Score on test b. PCPs care practices c. Descriptive information on delivered care	• Pre-test and post-tests • Traditional and web-based questionnaires • Web-based anonymous partial patient records
**4b) attitudes toward patients and treatment effectiveness**	a. Score on test b. Qualitative assessment of attitudes	• Pre-test and post-tests • Traditional and web-based questionnaires • Focus groups
**4c) perception of self-efficacy**	Qualitative assessment of perception	• Traditional and web-based questionnaires • Focus groups
**5. **Deliver generalizable and transferable knowledge on		
**5a) **the potential pitfalls and strategies of setting up preceptorships/website/virtual community network	Obstacles and facilitating factors in implementing the program	• Focus groups with participants, secondary care team members & decision-makers
**5b) **appropriate performance indicator sets assessing obesity care accessibility and delivery	Performance indicators	

### Data analysis and variable definition

Data from different sources will be grouped according to the evaluation questions. This triangulation will allow validation of the information gleaned from the different sources. Regarding quantitative data, appropriate descriptive statistics will be used to present the results, i.e. means with standard deviations for normally distributed variables, median with inter-quartile ranges for variables not distributed normally, and proportions with 95% confidence intervals for categorical variables. These descriptive statistics will be of prime importance to assess conformity with accepted norms in the literature and norms considered acceptable for each target group and decision makers.

Subgroup analyses will be performed to compare for example utilization, satisfaction or perceived utility regarding optimal care delivery among different web-based tools; satisfaction of all participants regarding preceptorship, web-site and virtual community; scores on the different tests before and after preceptorship and after one year of utilization of web-based tools and virtual-community; etc. Sub-group analyses will be performed using ANOVA tests and repeated-measures ANOVA tests, with HSD Tukey multiple comparison tests.

Qualitative data will be analyzed using codification of all comments and information. Coded informations will be grouped under specific themes or concepts. Proportions of reported themes or concepts for each question or subject of discussion will be reported. These data will be particularly useful to develop and improve the program under objectives 1 and 2, and to assess pitfalls and strategies for implementation of the program in general settings (objective 5). Qualitative data are also of prime importance to interpret and understand results obtained from typical quantitative data, and generate hypotheses for future studies.

### Significance, Impact and transferability

This research program will deliver transferable knowledge on management of obesity to primary care givers and will identify the potential pitfalls and strategies of setting up preceptorships and integrated interdisciplinary primary care team networks. Importantly, it will also yield important information on appropriate performance indicator sets for the future assessment of care access and delivery. The data derived from our study could be highly useful to health-system managers in Canada in order for them to implement truly integrated primary care delivery systems and programs targeting the obesity epidemic that we are facing. Date on financial and human resource requirements will be important for their planning.

The process and outcome measures are relevant and useful to a number of health system managers and policy makers. The *ADRLSSSS *of Estrie and Saguenay regions, as well as stakeholders from FMGs and the Public Health Directorate, will be actively involved in this research proposal. Our results will impact on the planning, allocation and management of resources as they apply to service organization. Early involvement of decision makers as co-investigators and active participants in our research project considerably increase the likelihood of implementation of changes in our health care system based on the outcomes of the present proposal. Research results are anticipated to be applicable to other institutions and regions of Quebec and Canada because most regions have access to specialists who could support networking with primary care teams. Our evaluation will also allow us to improve our program and, therefore, will also benefit our local health care system.

### Knowledge translation and transfer plan

It is worth mentioning that both the research program and the research design of this study are optimal to allow knowledge translation (KT) and application throughout the entire research process. Participants in the study will play an active role in validating the educational and clinical tools, discussing themes, applying new knowledge in their practice, and giving and receiving feedback during interdisciplinary and disciplinary team meetings and focus groups. Importantly, the preceptorship experience in itself is a knowledge translation activity for health professionals.

However, research by itself does little to induce change (except for participants) and thus, results have to be diffused to interested audiences. We have thus reserved a seven-month period exclusively aimed at knowledge transfer. We have access to specialized resources in communication from the Centre de recherché clinique Étienne-Le Bel to develop an optimal strategy based on KT literature [46-51].

Our research is relevant to: PCPs, nurse coordinators, specialists in various disciplines and associated health professionals, CME departments, public health directorates, health-system decision-makers. The research team members will thus reach out to these parties through linkage and exchange activities, including presentation of study results at scientific meetings and publication in scientific journals, but also by direct reports to decision-makers and health policy stakeholders. A clear summary of research results, including key messages targeted for each selected audience and synthesized results (divided by themes) will be made available in print and on a web site. Local media will be invited to a press conference. These approaches will first be pilot tested and should increase the use and widen the application of our results.

Our research team is composed of members that have preferred access to various interest groups. The decision-maker co-applicants will be instrumental in making contacts with other decision-makers at the provincial and national levels in order to eventually have an impact on health care organization. As already mentioned, the probability is high that our decision-maker co-investigators will appropriate and make use of the results of this study, because they will be involved early and actively in the research. Other members will easily reach specialists, primary care physicians, nurse-coordinators, public health stakeholders, CME departments and organizations; these interventions could impact obesity care delivery and CME delivery.

The results of this study will also be disseminated in medicine, nursing and other health professional discipline curricula to implicate them at an early stage in their training programs; Faculties of Medicine and Health Sciences will be invited to a web-based presentation of our results. The interdisciplinary-based practice that prevails in the study will be proposed to graduate students as an example of a methodological approach to program evaluation and implementation. Interest groups and community support group will also benefit from website conferences and workshops on the results of this study.

## Discussion

This project is relevant to health system's decision-makers who are confronted with an important increase in the prevalence of obesity. It is therefore critical to develop strategies allowing the management of obesity in the 1^st ^line setting. Results of this research project could therefore influence health care organization in the field of obesity but also eventually for other chronic diseases.

Should our data show that this integrated obesity care management system is effective in modifying short and long-term outcomes, future studies could concentrate on evaluating health indicators and patient satisfaction with such a system. The development of additional electronic tools for direct patient use may also turn out to be necessary. Other avenues of research include assisting other teams and decision makers in the implementation of similar programs or other research projects that will arise from our knowledge transfer activities.

In view of the limited resources (financial and time) available for the realization of the proposed project, we will not be able to measure the clinical outcomes of patients followed by the research participants. However, we view this application as a first step in the study of the impact of our integrated obesity care system on participating health professionals and their practice. Therefore the study of clinical outcomes of patients cared for by participants in a program, such as the one proposed here, compared to a control group of patients cared for by non-participating physicians, will be the object of a future and grant application.

## Competing interests

The author(s) declare that they have no competing interests.

## Authors' contributions

All authors read and approved the final manuscript.

## Pre-publication history

The pre-publication history for this paper can be accessed here:


